# The YHS-Domain of an Adenylyl Cyclase from *Mycobacterium phlei* Is a Probable Copper-Sensor Module

**DOI:** 10.1371/journal.pone.0141843

**Published:** 2015-10-29

**Authors:** Jürgen Ulrich Linder

**Affiliations:** Department of Biochemistry, University of Bayreuth, Bayreuth, Germany; University of Saskatchewan, CANADA

## Abstract

YHS-domains are small protein modules which have been proposed to bind transition-metal ions like the related TRASH-domains. They are found in a variety of enzymes including copper-transporting ATPases and adenylyl cyclases. Here we investigate a class IIIc adenylyl cyclase from *Mycobacterium phlei* which contains a C-terminal YHS-domain linked to the catalytic domain by a peptide of 8 amino acids. We expressed the isolated catalytic domain and the full-length enzyme in *E*. *coli*. The catalytic domain requires millimolar Mn^2+^ as a cofactor for efficient production of cAMP, is unaffected by low micromolar concentrations of Cu^2+^ and inhibited by concentrations higher than 10 μM. The full-length enzyme also requires Mn^2+^ in the absence of an activator. However, 1–10 μM Cu^2+^ stimulate the *M*. *phlei* adenylyl cyclase sixfold when assayed with Mn^2+^. With Mg^2+^ as the probable physiological cofactor of the adenylyl cyclase Cu^2+^ specifically switches the enzyme from an inactive to an active state. Other transition-metal ions do not elicit activity with Mg^2+^. We favor the view that the YHS-domain of *M*. *phlei* adenylyl cyclase acts as a sensor for copper ions and signals elevated levels of the transition-metal via cAMP. By analogy to TRASH-domains binding of Cu^2+^ probably occurs via one conserved aspartate and three conserved cysteine-residues in the YHS-domain.

## Introduction

Cellular signal transduction is achieved by a complex molecular network to enable the cell to adapt to and react to changes in its environment. Many signal transduction processes involve second messengers. Upon activation of a single second messenger generating protein many of these signaling molecules are produced thereby amplifying the original input signal.

A central second messenger in eukaryotic as well as prokaryotic signal transduction is 3’,5’-cyclic adenosine monophosphate (cAMP). cAMP is generated from ATP by adenylyl cyclases. To date six classes of adenylyl cyclases (ACs) have been described which do not share any sequence similarity and are thought to be the product of convergent evolution [1–4]. By far the largest number of ACs belongs to class III. Class III ACs are found in metazoans including mammals, in protozoans and in eubacteria [1].

The catalytic domain of class III ACs is often termed CHD (cyclase homolgy domain). It forms head-to-tail dimers with the catalytic centers located at the interface of the dimer, thus making dimerization a prerequisite for catalytic activity [5,6]. Based on analysis of amino acid sequences and structures of their catalytic domains the class III ACs have been classified in four categories, class IIIa through class IIId [7]. According to the Interpro protein domain organisation database almost all class III ACs are multi-domain proteins [8]. The general role of many additional domains is that of signal receivers. A stimulus acting on those domains is transmitted to the catalytic domain, usually leading to activation of the CHD [9–12]. Because often regulatory domains associated with ACs are also occuring in other families of signal transduction proteins, the investigation of such ACs can greatly further our understanding of these domains in a more general context [9,12,13].

**Fig 1:**
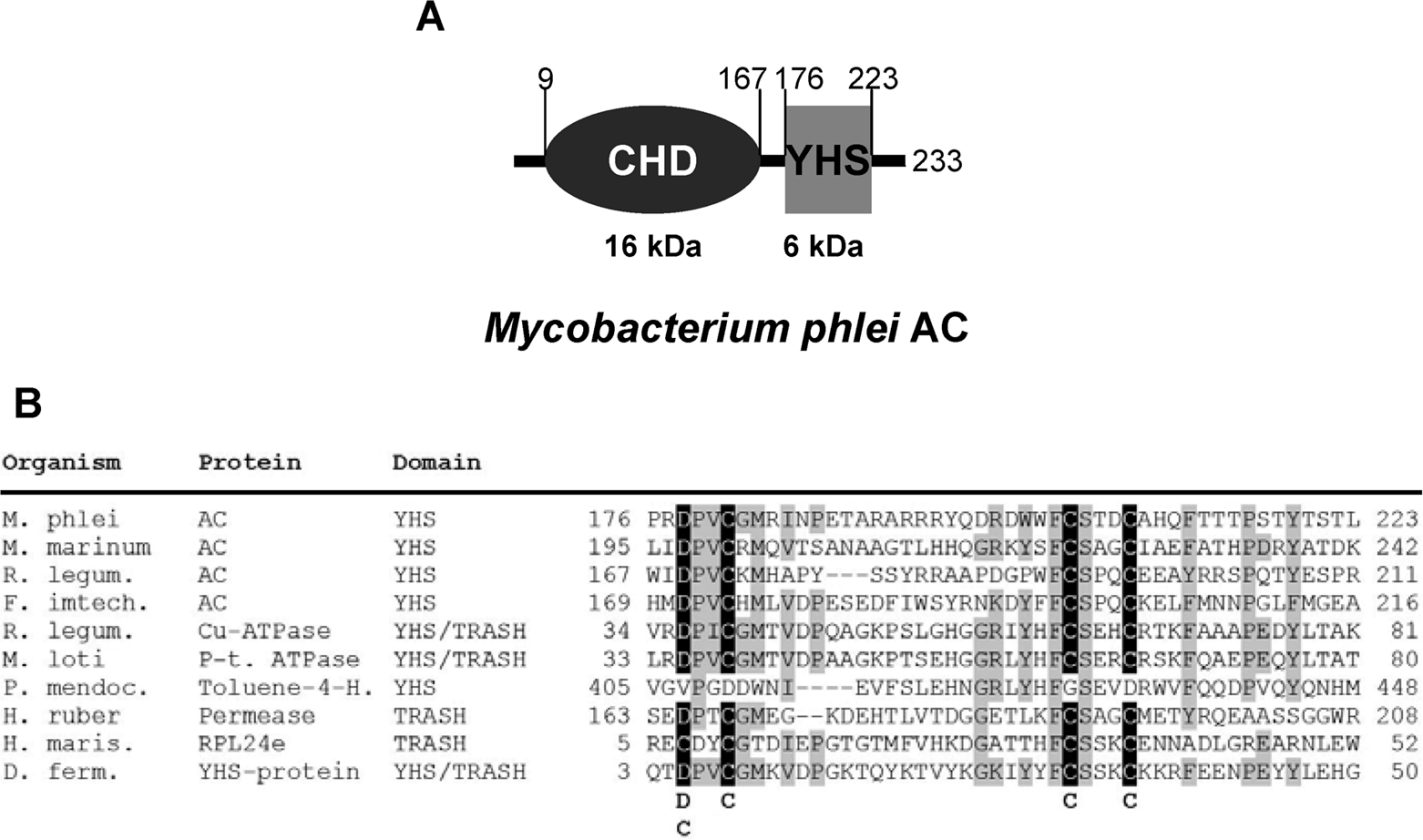
Sequence analysis of YHS-domains in ACs. (A) Modular organisation of ACs containing YHS-domains. Amino acid numbering refers to the AC from *M*. *phlei*. (B) Alignment of YHS-, YHS/TRASH- and TRASH-domains from various bacterial proteins. The four conserved residues implicated in transition-metal binding are shaded black. Other conserved residues are shaded grey. *M*. *phlei* AC, GenBank EID14989.1; *Mycobacterium marinum* AC, GenBank ACC39874.1; *Rhizobium leguminosarum* AC, GenBank KEC71354.1; *Fulvivirga imtechensis* AC, GenBank ELR73472.1; *Rhizobium leguminosarum* Cu-ATPase, GenBank AAD26860.1; *Mesorhizobium loti* P-type ATPase, GenBank BAB51797.1; *Pseudomonas mendocina* toluene-4-hydroxylase, GenBank AAA25999.1; *Halovivax ruber* permease, GenBank AGB15007.1; *Haloarcula marismortui* ribosomal protein L24e, GenBank AAV45180.1; *Desulfurococcus fermentans* YHS-protein, GenBank AFL66382.1.

In the present study we explored the function of the YHS-domain. The YHS-domain is a small cytosolic protein domain of ca. 50 amino acids named after three conserved amino acid residues, *i*.*e*. tyrosine, histidine, serine. It has first been published as part of the InterPro database (InterPro IPR007029). The YHS-domain shares extensive similarity to the TRASH domain, a protein domain binding transition-metal ions via conserved cysteine residues [14]. Due to the similarity of YHS to TRASH many domains in InterPro are annotated as both, YHS and TRASH (Fig 1B). Thus, the YHS-domain may be regarded as an expansion of the TRASH domain.

Because of its occurrence in a number of bacterial Cu-ATPases it has been proposed that the YHS-domain binds copper ions. However, binding of copper ions to YHS/TRASH has not been experimentally demonstrated in these transporters. A YHS-domain is also found in toluene-4-hydroxylase. The crystal structure of this enzyme has been solved including the YHS-domain [15]. However, neither a metal ion nor any other ligand is bound to YHS in the crystal. Furthermore copper ions have not been implicated in the catalytic mechanism or regulation of the hydroxylase and the function of the YHS-domain is unknown. The prokaryotic ribsomal protein L24e consists of a single TRASH domain. In the crystal structure of the 50S ribosome from H. marismortui [16], a Cd^2+^-ion is bound to L24e via the conserved cysteine residues giving experimental evidence of a transition-metal ion binding TRASH-domain and thus corroborating the proposed function of YHS-domains.

YHS-domains are also present in about 20 putative class III adenylyl cyclases, predicted from whole genome sequencing data. They occur mainly in mycobacterial species, but also in diverse eubacteria like *Gordonia bronchialis* and several *Rhizobium* species. In these ACs the CHD is fused to the YHS-domain located at the C-terminal end.

Here we expressed, purified and characterized the YHS-domain containing AC from *Mycobacterium phlei*. We show that the enzyme is an active AC and is activated by copper ions. Activation relies on the presence of the YHS-domain. Thus the AC-associated YHS-domain appears to act as a sensory module for copper ions.

## Materials and Methods

### Cloning of *Mycobacterium phlei* AC

The gene for *M*. *phlei* AC (MpAC, GenBank accession EID14989.1) was codon-optimized for expression in *E*. *coli* and fitted with an N-terminal *Bam*HI and a C-terminal *Hind*III site (S1 Fig). The DNA was synthesized commercially (GeneArt/life technologies). For expression of MpAC_1-182_ the synthetic gene was transfered via the respective *Bam*HI and *Hind*III sites into the vector pQE30 (Qiagen). The YHS-domain was then removed by cutting with *Sph*I and *Hind*III, blunting the ends by treatment with *Klenow*-enzyme and recircularization of the product. The resulting open reading frame codes for MRGSH_6_GS-MpAC_1-182_-A. For expression of the full-length enzyme the gene was cloned into the *BamH*I and *Hind*III sites of a modified pQE30-vector coding for a TEV-protease recognition sequence downstream of the hexahistidine motif. The open reading frame codes for MRGSH_6_GSENLYFQGS-MpAC_1-233_ (His-TEV-MpAC_1-233_). Expression plasmids were sequenced for checking the intactness of the expression cassettes.

### Expression and purification of *M*. *phlei* adenylyl cyclase

MpAC was produced in *E*. *coli* BL21(DE3)[pRep4]. Batch size was 1 liter of LB-medium supplied with 100 mg/l ampicillin and 25 mg/l kanamycin. Cells were induced with 0.1 mM isopropyl-1-thio-β-D-galactopyranoside for 5–6 hrs at 20°C. Bacteria were washed with buffer (50 mM Tris/HCl, 1 mM EDTA, pH 8), frozen in liquid nitrogen and stored at -80°C. Purification was started by suspending the cells in 20 ml lysis buffer (50 mM Tris/HCl pH 8), sonicating for 40 s and treating with 0.2 mg/ml lysozyme for 30 min on ice. After addition of 5 mM MgCl_2_ and 20 μg/ml of DNaseI the incubation was continued for another 30 min. After centrifugation (31000 x *g*, 30 min) the solution was supplemented with 250 mM NaCl and 15 mM imidazole (final concentrations). 600 μl Ni-NTA-agarose were added and the mixture was gently shaken for 3 hours on ice. The resin was transferred into a column, washed with 10 ml buffer A (lysis buffer containing 250 mM NaCl, 15 mM imidazole, 5 mM MgCl_2_) and subsequently with 5 ml of buffer B (lysis buffer containing 15 mM imidazole, 5 mM MgCl_2_). Protein was eluted with 0.6 ml of buffer C (37.5 mM Tris/HCl, pH 8, 250 mM imidazole, 2 mM MgCl_2_). Purified MpAC was stored at -20°C after addition of 40% glycerol. The purity of the recombinant proteins was assessed by 14% SDS-PAGE and staining with Coomassie Blue G250.

### Removal of the hexahistidine-tag of full-length MpAC

Purified His-TEV-MpAC_1-233_ was diluted with an equal volume of 20 mM Tris/HCl, pH 8 and 0.12 μg His-tagged TEV-protease per μg AC was added. The mixture was incubated for 12 hours at 4°C. Buffer was rapidly changed by consecutive dilution and ultrafiltration to 20 mM Tris/HCl, pH8; 7 mM Imidazole; 50 mM NaCl; 2 mM MgCl_2_. 250 μl Ni-NTA-Agarose per 100 μg of protein were added and incubation was performed for 3 hours on ice. The resin was removed by filtration. The filtrate was mixed with 25% glycerol and stored at -20°C. The final product carries an N-terminal dipeptide compared to native MpAC. The sequence is GS-MpAC_1-233_.

### AC assay

AC activity was measured for 10 min at 30°C in a volume of 30 μl. Standard reactions contained 2.5 mM Tris/HCl, pH 8.0, 5 mM MnCl_2_ or MgCl_2_, 1 mM ATP. cAMP was determined by HPLC as described previously [17]. All data are means of 2 to 6 points and are denoted with their standard deviations.

### Tryptophane-fluorescence spectroscopy

Samples were irradiated in a 1 ml fluorescence cuvette at 295 nm (gap width 5 nm) at 20°C. Fluorescence intensities at 345 nm were recorded with a gap width of 5 nm. Fluorescence data were corrected for the inner-filter effect of protein and reagents and for the dilution factor upon addition of reagents.

## Results and Discussion

### Primary structure of ACs containing a YHS domain

A search of the InterPro protein domain architecture database [8] for protein sequences containing class III adenylyl cyclase catalytic domains (CHDs, InterPro IPR001054) yielded a list of 1369 different architectures present in a total of 19961 protein sequences. Among these, 13 proteins were found which contained a YHS domain. All share the same architecture of a single CHD linked to a single C-terminal YHS domain (Fig 1A). The linker region between the two domains consists of 8–11 residues. Using the AC-YHS protein from *Mycobacterium phlei* (GenBank accession EID14989.1) as a query, a BLAST search of the non-redundant protein data base [18] yielded a set of 24 sequences, partially overlapping the results from the InterPro search. A total number of 26 putative AC-YHS enzymes were detected by the combination of both searches. A representative sequence alignment is given in the supplementary material (S2 Fig).

We have focused our work on the AC form *M*. *phlei* because of its compact structure with 233 residues compared to a range of 216–473 amino acids (aa) among all AC-YHS sequences and because *M*. *phlei* is a well characterized species with a history as a mycobacterial model organism [19]. Inspection of the CHD of the *M*. *phlei* AC revealed that all six canonical catalytic residues [5,7] are present (highlighted in S2 Fig). Furthermore the dimerization arm of the *M*. *phlei* AC is short with 7 residues, which classifies it as a class IIIc CHD [7]. Analysis of the complete set of AC-YHS proteins shows that all of them belong to that subclass.

An alignment of the YHS domains of ACs with the YHS/TRASH domains of Cu-ATPases and other YHS and TRASH domains is shown in Fig 1B. The mode of metal-ion coordination has previously been uncovered by the crystal structure of the ribosomal TRASH-domain subunit L24e [16]. A Cd^2+^ ion is bound to four cysteine residues which are highlighted in Fig 1B. In the YHS domain of ACs the N-terminal cysteine is replaced by aspartate (D178 in *M*. *phlei* AC), while the other three cysteine residues are conserved (*M*. *phlei* AC: C181, C204, C208). An aspartate in the position of the first metal-ion coordinating residue appears to be common among YHS and TRASH-domains (Fig 1B). In general the sequence profile of the YHS-domains of bacterial ACs fits that of bacterial Cu-ATPases and other metal-ion binding YHS- and TRASH-domains, which led us to the hypothesis that the AC of *M*. *phlei* may bind transition-metal ions, probably copper, via its YHS-domain and that it may be regulated by such ions.

### Expression and characterization of the catalytic domain of *M*. *phlei* adenylyl cyclase

**Fig 2:**
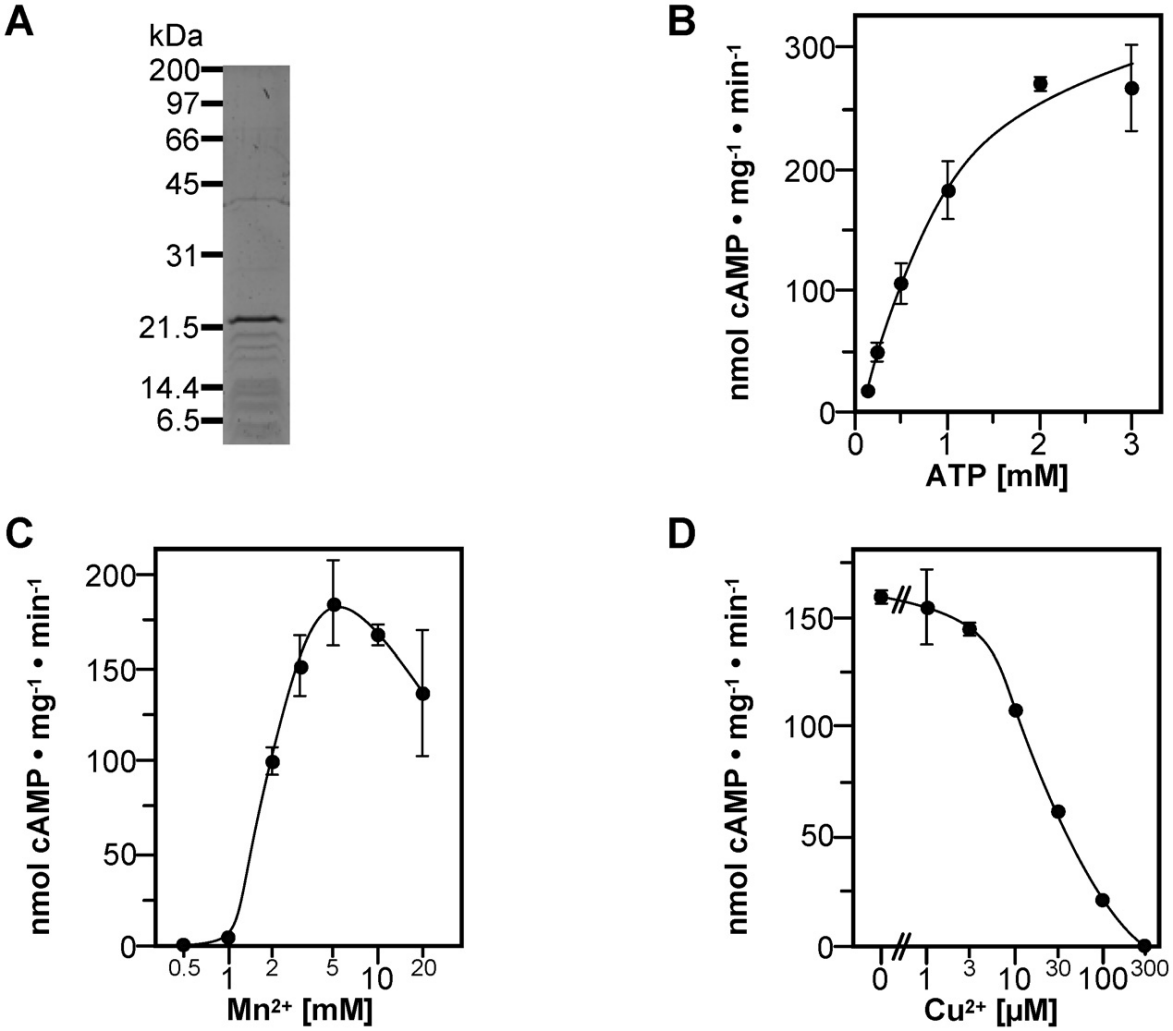
Biochemical analysis of the catalytic domain MpAC_1-182_. (A) SDS-PAGE of MpAC_1-182_. (B) Substrate kinetics measured with 5 mM Mn^2+^ as a cofactor at 30°C and pH 8.0. Vertical bars indicate the standard deviation if larger than symbol size. (C) Mn^2+^-dependance assayed at 1 mM ATP. (D) Inhibition by Cu^2+^. Reactions contained 1.6 μM MpAC_1-182_, 5 mM Mn^2+^ and 1 mM ATP.

In a first step towards investigation of the YHS-domain the isolated catalytic domain of *M*. *phlei* AC (MpAC) was characterized to provide a base for the separation of direct effects of metal-ions on the CHD from effects mediated by the YHS-domain. The CHD (aa 1–182) of MpAC was N-terminally tagged with a hexahistidine motif and expressed in *E*. *coli*. Upon purification of MpAC_1-182_ by affinity chromatography the protein migrated at 22 kDa on SDS-PAGE (calculated 20.1 kDa, Fig 2A). The identity of the protein was confirmed by mass spectrometry of a tryptic digest (data not shown). MpAC_1-182_ showed robust AC activity of 196 ± 12 nmol cAMP mg^-1^ · min^-1^ with Mn^2+^ as a cofactor whereas activity with Mg2+ was minimal with 0.37 ± 0.02 nmol cAMP mg^-1^ · min^-1^. With Mn^2+^ as a cofactor MpAC_1-182_ displayed maximal activity in the range of pH 8.0–8.5. The temperature optimum was at 30°C (data not shown). Subsequently all assays were performed at 30°C and pH 8.0.

Kinetic analysis showed a v_max_ of 335 ± 70 nmol cAMP mg^-1^ · min^-1^ with half-maximal activity (SC_50_) at 0.90 ± 0.22 mM ATP and a pronounced positive cooperativity for ATP as seen by a Hill coefficient of 1.5 ± 0.2 (Fig 2B, Table 1). The Mn^2+^-dependence of MpAC_1-182_ at 1 mM ATP is shown in Fig 2C. Activity is low up to 1 mM Mn^2+^ and surging when the concentration of Mn^2+^ exceeds that of ATP. This behavior is typical for the two-metal ion mechanism of ACs and demonstrates the requirement of both, MnATP and free Mn^2+^ for catalysis to occur [20]. Based on a reciprocal plot the affinity of MpAC_1-182_ for the cofactor was calculated yielding an EC_50_ for free Mn^2+^ of 2.0 ± 0.6 mM.

**Table 1:** Kinetic parameters of MpAC_1-182_ and MpAC_1-233_.

Enzyme	V_max_ [nmol cAMP · mg^-1^ · min^-1^]	SC_50_ [mM ATP]	Hill-coefficient	EC_50_ [mM Me^2+^]
MpAC_1-182_/ Mn^2+^	335 ± 70	0.90 ± 0.22	1.5 ± 0.2	1.95 ± 0.58
MpAC_1-233_/ Mn^2+^	1660 ± 90	2.11 ± 0.54	0.9 ± 0.1	8.27 ± 0.57
MpAC_1-233_/ Mn^2+^/Cu^2+^	3630 ± 1060	0.37 ± 0.15	1.2 ± 0.3	0.68 ± 0.17
MpAC_1-233_/ Mg^2+^/Cu^2+^	485 ± 35	1.63 ± 0.23	1.5 ± 0.1	1.84 ± 0.82

Reactions were performed under standard-conditions (30°C, pH 8.0) with 5 mM cofactor (Mn^2+^ or Mg^2+^) and with addition of 10 μM Cu^2+^ where indicated. EC_50_ values for the cofactor ion were determined at 1 mM ATP. Values are means ± SD, n = 2–4.

Because copper ions are activators of full-length MpAC (see below), the effect of Cu^2+^ on MpAC_1-182_ was tested (Fig 2D). Cu^2+^ acted as an inhibitor of the CHD with an IC_50_ of 20 μM.

### Expression and characterization of full-length *M*. *phlei* adenylyl cyclase

**Fig 3:**
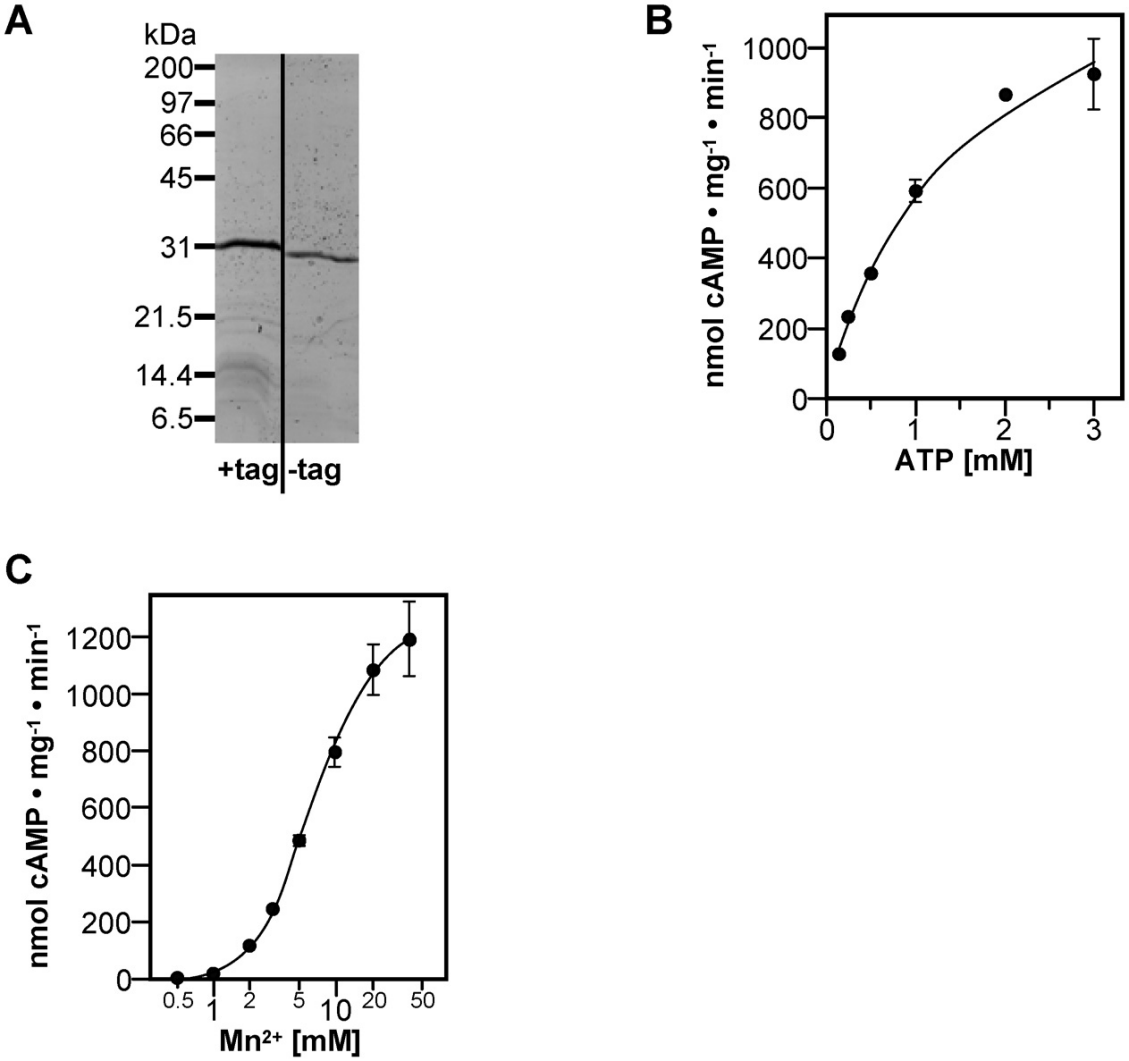
Biochemical analysis of full-length MpAC_1-233_. (A) SDS-PAGE of His-TEV-MpAC_1-233_ (+tag) and untagged MpAC_1-233_ (-tag). Both lanes are from the same gel and are shown in alignment with the molecular weight marker on that gel. (B) Substrate kinetics measured with 5 mM Mn^2+^ as a cofactor at 30°C and pH 8.0. (C) Mn^2+^-dependance assayed at 1 mM ATP.

The full-length MpAC was fitted with an N-terminal hexahistidine-tag carrying a TEV-protease recognition sequence and expressed in *E*. *coli*. Purified His-TEV-MpAC_1-233_ appeared at 31 kDa on SDS-PAGE, slightly higher than expected (calculated 27.0 kDa, Fig 3A). Subsequently the hexahistidine-tag was removed with TEV protease and the untagged full-length *M*. *phlei* AC was purified by reverse Ni^2+^-affinity chromatography. Untagged MpAC_1-233_ showed a slightly higher mobility on SDS-PAGE compared to His-TEV-MpAC_1-233_ demonstrating the success of the tag-removal procedure (Fig 3A).

The specific activity of MpAC_1-233_ with Mn^2+^ as a cofactor was 541 ± 56 nmol cAMP mg^-1^ · min^-1^, while no activity was detectable with Mg^2+^. The v_max_ of 1660 ± 90 nmol cAMP mg^-1^ · min^-1^ and SC_50_ of 2.11 ± 0.54 mM ATP were higher than the corresponding values of the isolated CHD (Fig 3B, Table 1). In addition, MpAC_1-233_ did not show cooperativity with a Hill coefficient of 0.9 ± 0.1. The Mn^2+^-dependance of MpAC_1-233_ showed a similar requirement for Mn^2+^ in excess of ATP like that of MpAC_1-182_. Yet, the affinity for Mn^2+^ was lower for MpAC_1-233_ with an EC_50_ of 8.3 ± 0.6 mM free Mn^2+^ (Fig 3C). The change in the kinetic parameters and the Mn^2+^-affinity by the presence of the YHS-domain indicates that the YHS-domain has an impact on the conformation of the CHD even in the absence of transition-metal ions.

### Stimulation of full-length *M*. *phlei* adenylyl cyclase by copper ions with Mn^2+^ as a cofactor

**Fig 4:**
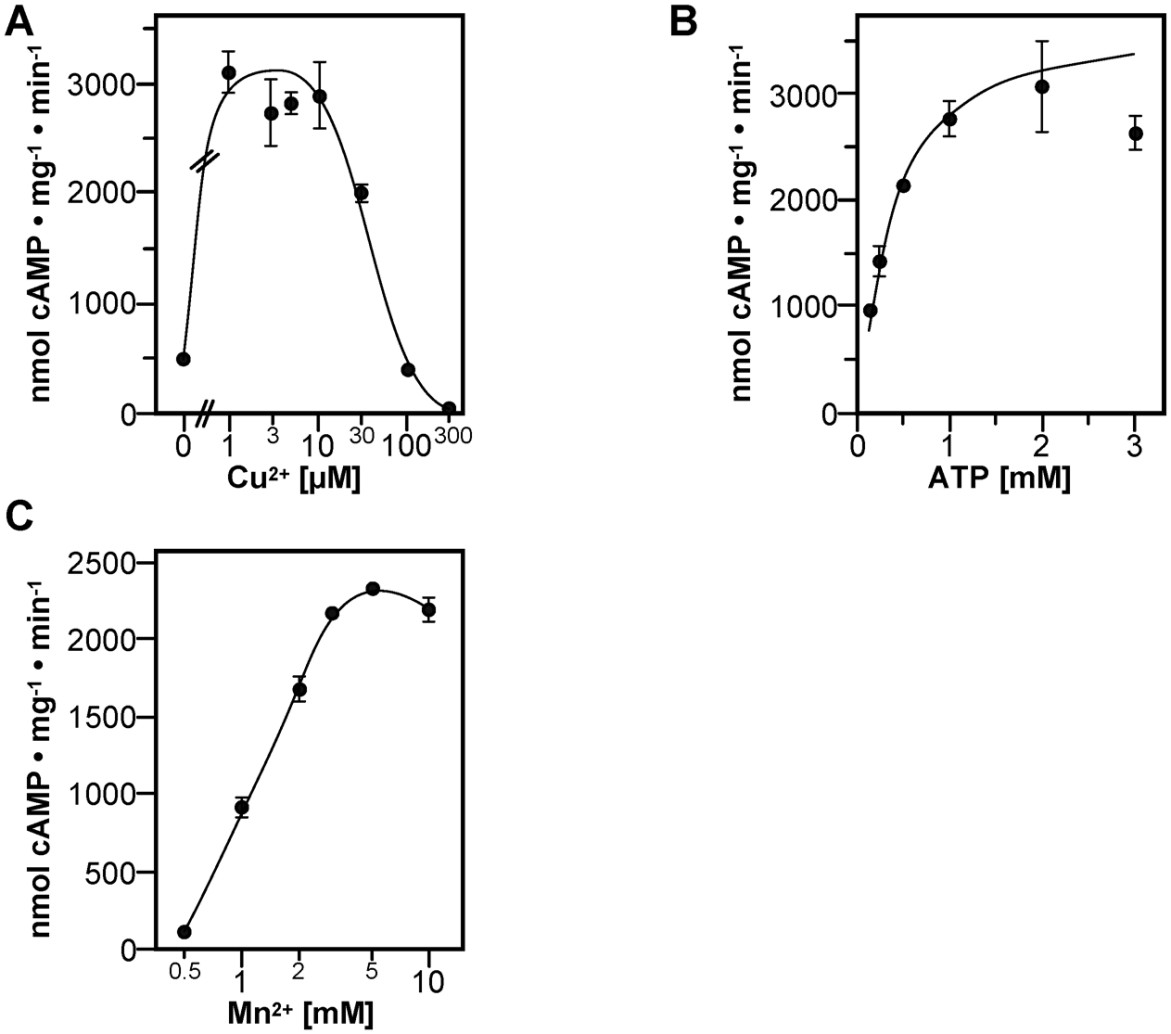
Stimulation of full-length MpAC_1-233_ by Cu^2+^ assayed with Mn^2+^ as a cofactor. (A) Effect of Cu^2+^. Reactions contained 0.4 μM MpAC_1-233_, 5 mM Mn^2+^ and 1 mM ATP. (B) Substrate kinetics measured with 5 mM Mn^2+^ as a cofactor and 10 μM Cu^2+^. (C) Mn^2+^-dependance assayed at 1 mM ATP and 10 μM Cu^2+^.

The hypothesis of the YHS-domain of *M*. *phlei* AC being a transition-metal ion binding domain with similarity to the YHS-domains of CuATPases was tested in assays of the untagged full-length MpAC_1-233_ with micromolar concentrations of Cu^2+^ and Mn^2+^ as a cofactor (Fig 4A). Cu^2+^ maximally stimulated MpAC_1-233_ sixfold at concentrations of 1 to 10 μM. Because we used an enzyme concentrations of 0.4 μM to assure efficient dimerization of MpAC_1-233_ (see below), assays with Cu^2+^ below 1 μM were not conducted. Yet, the data imply that the EC_50_ for activation by Cu^2+^ is in the nanomolar range. On the other hand, high concentrations of Cu^2+^ were inhibitory similar to the results seen with the isolated catalytic domain. Thus, we concluded that the stimulatory effect of low concentrations of Cu^2+^ on MpAC_1-233_ is mediated by the YHS-domain. The inhibitory effect of higher concentrations of Cu^2+^ may be due to general interactions and reactions with protein similar to those exploited in classic protein assays like the Lowry- and the Biuret-method [21]. Kinetic analysis of MpAC_1-233_ showed that 10 μM Cu^2+^ led to an increase in v_max_ and a concomitant decrease of SC_50_ for ATP (Fig 4B, Table 1). The Hill coefficient of 1.2 ± 0.3 indicated that Cu^2+^ did not induce pronounced cooperativity for ATP. Furthermore, addition of 10μM Cu^2+^ resulted in a 10-fold higher affinity for the cofactor Mn^2+^ with an EC_50_ of 0.7 ± 0.2 mM for the free ion (Fig 4C). Taken together Cu^2+^ appears to increase the catalytic efficiency of the enzyme.

### Activation of full-length *M*. *phlei* adenylyl cyclase by copper ions with Mg^2+^ as a cofactor

Because a requirement of millimolar concentrations of Mn^2+^ for AC activity may not reflect the physiological environment of the enzyme in *Mycobacterium phlei* we explored the effect of Cu^2+^ on MpAC_1-233_ with Mg^2+^ as a cofactor. MpAC_1-233_ produced 122 ± 20 nmol cAMP mg^-1^ · min^-1^ upon addition of 10 μM Cu^2+^. Thus Cu^2+^ served as an on-switch of MpAC that relies on the YHS-domain of the enzyme.

**Fig 5:**
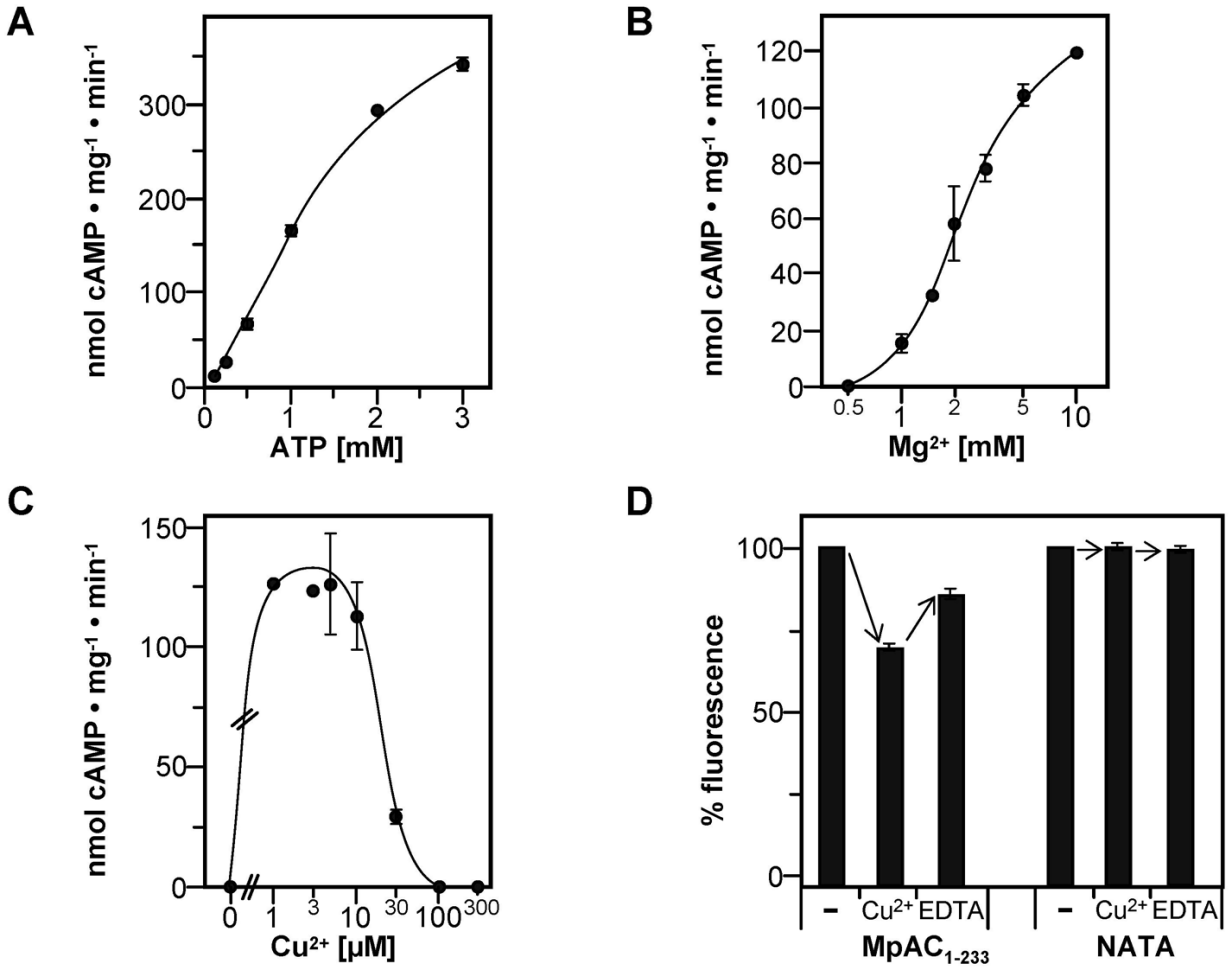
Activation of full-length MpAC_1-233_ by Cu^2+^ assayed with Mg^2+^ as a cofactor. (A) Substrate kinetics measured with 5 mM Mg^2+^ as a cofactor and 10 μM Cu^2+^. (B) Mg^2+^-dependance assayed at 1 mM ATP and 10 μM Cu^2+^. (C) Effect of Cu^2+^. Reactions contained 0.4 μM MpAC_1-233_, 5 mM Mg^2+^ and 1 mM ATP. (D) Tryptophane-fluorescence spectroscopy. The fluorescence emission signal at 345 nm of 1.15–2.55 μM MpAC_1-233_ (n = 3) or 5 μM N-acetyl-tryptophaneamide (NATA, n = 2) was taken as 100% (-). Subsequently 25 μM Cu^2+^ were added (Cu^2+^) and finally the copper-ions were complexed with 375 μM EDTA (EDTA).

Kinetic analysis (Fig 5A) yielded a v_max_ of 485 ± 35 nmol cAMP mg^-1^ · min^-1^ with an SC_50_ of 1.63 ± 0.23 mM ATP. The Hill coefficient of 1.5 ± 0.1 demonstrated a positive cooperativity for ATP with Mg^2+^ in contrast to assays with Mn^2+^ (Table 1). Mg^2+^-dependance showed the typical characteristics of a two-metal ion mechanism as seen with Mn^2+^; EC_50_ for free Mg^2+^ was 1.8 ± 0.8 mM (Fig 5B). Activation by Cu^2+^ was maximal in the range of 1 to 10 μM with strong inhibition at higher concentrations (Fig 5C).

If the YHS-domain served as a Cu^2+^-sensor the activation of MpAC_1-233_ should be specific to copper ions. MpAC_1-233_ was assayed with Mg^2+^ as a cofactor and addition of Zn^2+^, Fe^2+^, Fe^3+^, Cr^3+^, Co^2+^ and Ni^2+^. There was no detectable AC activity with any of these transition-metal ions. Next we checked whether activation of MpAC_1-233_ by Cu^2+^ occurs by binding of the metal ion to the enzyme or whether it is a consequence of the oxidative properties of Cu^2+^, *e*.*g*. by oxidizing the conserved cysteine residues [22] of the YHS-domain. MpAC_1-233_ was incubated with 10 μM Cu^2+^ for 15 min in the presence of Mg^2+^ as a cofactor, then Cu^2+^ was selectively quenched by 375 μM EDTA before the reaction was started by addition of ATP. No AC activity was detected, while in a parallel experiment under same conditions, but without quenching by EDTA, AC activity was preserved (data not shown).

Finally, we investigated the effect of Cu^2+^ on the YHS-domain by tryptophane-fluorescence spectroscopy, which was facilitated by the only two tryptophane residues of MpAC_1-233_ being located there (W201 and W202). The fluorescence emission spectrum showed a maximum at 345 nm, which was unchanged by the addition of Cu^2+^ or EDTA (data not shown). However, the fluorescence intensity at 345 nm was significantly reduced by Cu^2+^ and addition of EDTA in essence reverted the effect (Fig 5D). As a control, the fluorescence of N-acetyl-tryptophanamide was unaffected by Cu^2+^ and EDTA showing that neither reagent acted as a general quencher of fluorescence (Fig 5D). The data are consistent with a conformational change of the YHS-domain triggered by the binding of Cu^2+^. Thus we favor the view that the *M*. *phlei* AC acts as a Cu^2+^-sensor enzyme, where binding of Cu^2+^ to the YHS-domain in turn activates the CHD.

Although we do not know the mechanism of activation by Cu^2+^ at this time, we can exclude an enhanced dimerization as an underlying principle. The dissociation constant of the enzyme is 0.16 μM as calculated from the protein dependance of the AC activity (data not shown). We used at least 0.4 μM MpAC_1-233_ in all assays to ensure that the enzyme largely remains dimerized. Crystal structures of class III ACs have shown that upon activation only minor changes in secondary structure occur, whereas often pronounced shifts in the orientation of the CHD monomers to each other are visible [5,10,17]. Similar mechanisms might trigger the activation of the *M*. *phlei* ACby Cu^2+^.

The physiological role of the Cu^2+^-sensor AC in the bacterium remains open to speculation. It has been known since the 1950’s that *M*. *phlei* is exquisitely sensitive to Cu^2+^ with strong growth inhibition occurring with as little as 1 μM Cu^2+^ in the medium [23]. For comparison 100 μM Cu^2+^ has no effect on the growth rate of *E*. *coli* [23]. A link between Cu^2+^-sensitivity and Cu^2+^-sensor AC in *M*. *phlei* therefore appears as a plausible hypothesis.

By structural analogy to the related TRASH-domain the conserved aspartate and cysteine residues marked in Fig 1B are likely to coordinate Cu^2+^ in the YHS-domain of *M*. *phlei* AC [14]. Our study provides biochemical evidence for a function of the YHS-domain in transition-metal ion binding and enzyme regulation which also impacts on our understanding of the TRASH-domain. Furthermore our data underline the high versatility of class III AC catalytic domains in their interaction with diverse regulatory domains as seen in past studies.

## Supporting Information

S1 Fig Sequence of the synthetic gene for Mycobacterium phlei AC. Flanking *Bam*HI and *Hind*III are shown in italics. Differences to the natural gene (GenBank EID14989.1) are underlined.
(TIF)

S2 Fig Alignment of ACs carrying a C-terminal YHS-domain. In the CHD, critical functional residues for metal-cofactor binding (M), adenine-moiety binding (A) and for catalysis (C) are shaded black, as well as the four conserved putative transition-metal ion binding residues of the YHS-domain. Sequences are from *Mycobacterium phlei* (GenBank EID14989.1), *Mycobacterium marinum* (GenBank ACC39874.1), Williamsia sp.ARP1 (NCBI reference WP_045825068.1); Candidatus *Microthrix parvicella* RN1; (GenBank CCM65060.1); *Rhizobium leguminosarum* (GenBank KEC71354.1); *Rhizobium etli* (NCBI reference WP_040141435.1); *Fulvivirga imtechensis* (GenBank ELR73472.1).
(TIF)
