# Supplementary figures and images for: The YHS-Domain of an Adenylyl Cyclase from Mycobacterium phlei Is a Probable Copper-Sensor Module
Source: PLoS One. 2015 Oct 29;10(10):e0141843. doi: 10.1371/journal.pone.0141843 (PMC4626032; doi:10.1371/journal.pone.0141843)

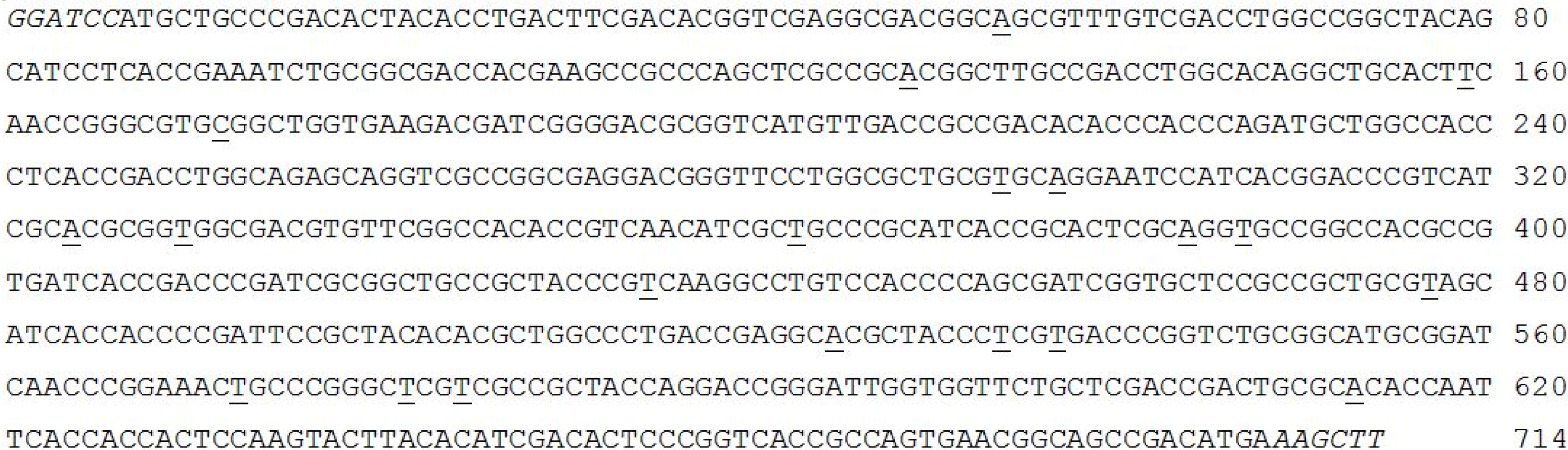

Supplement: S1 Fig — Flanking BamHI and HindIII are shown in italics. Differences to the natural gene (GenBank EID14989.1) are underlined. (TIF) [file pone.0141843.s001.tif]

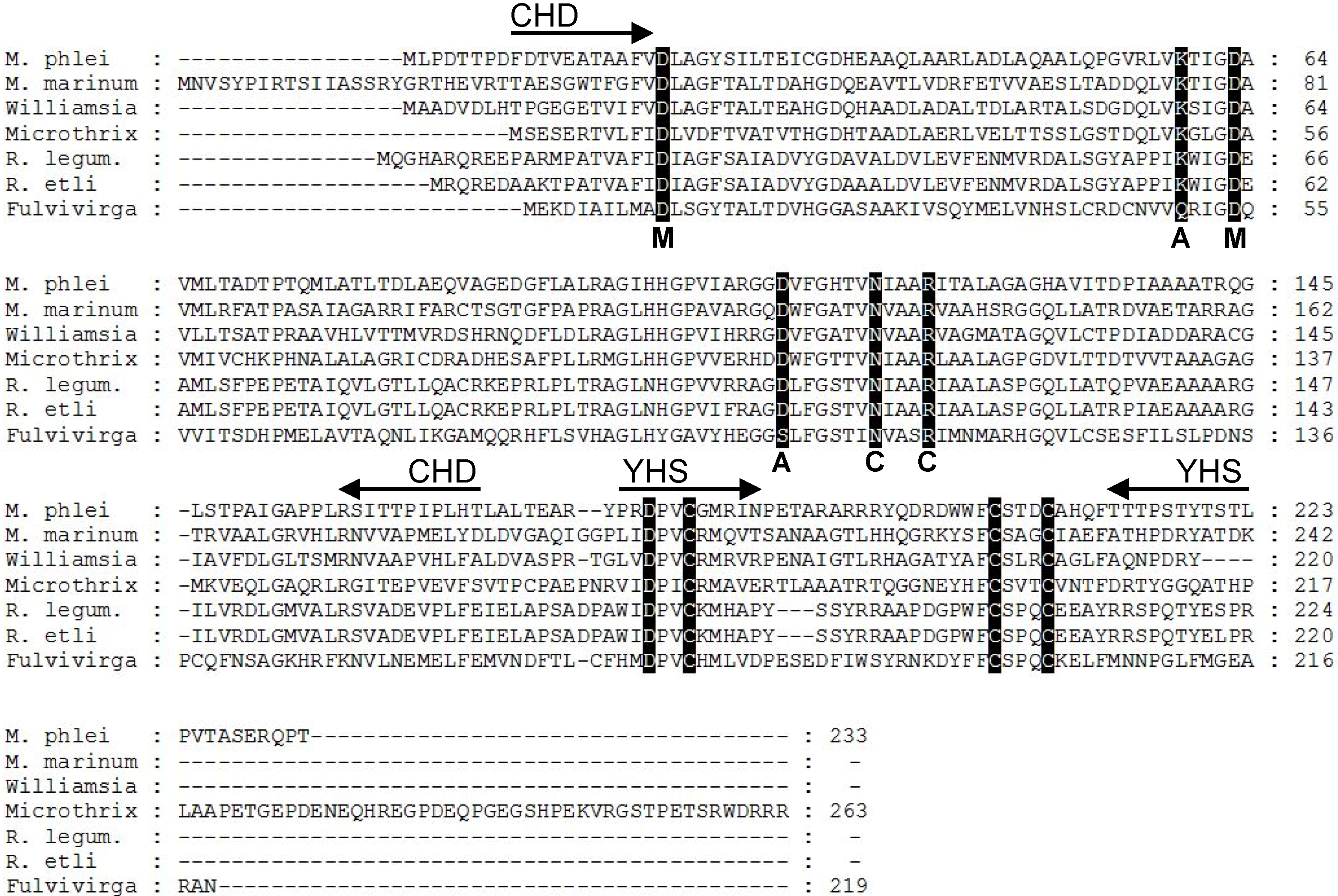

Supplement: S2 Fig — In the CHD, critical functional residues for metal-cofactor binding (M), adenine-moiety binding (A) and for catalysis (C) are shaded black, as well as the four conserved putative transition-metal ion binding residues of the YHS-domain. Sequences are from Mycobacterium phlei (GenBank EID14989.1), Mycobacterium marinum (GenBank ACC39874.1), Williamsia sp.ARP1 (NCBI reference WP_045825068.1); Candidatus Microthrix parvicella RN1; (GenBank CCM65060.1); Rhizobium leguminosarum (GenBank KEC71354.1); Rhizobium etli (NCBI reference WP_040141435.1); Fulvivirga imtechensis (GenBank ELR73472.1). (TIF) [file pone.0141843.s002.tif]
